# Conservative Otosclerosis Treatment With Sodium Fluoride and Other Modern Formulations: A Systematic Review

**DOI:** 10.7759/cureus.34850

**Published:** 2023-02-10

**Authors:** Panagiotis P Gogoulos, Giorgos Sideris, Thomas Nikolopoulos, Electra K Sevastatou, George Korres, Alexander Delides

**Affiliations:** 1 Second Ear, Nose, and Throat (ENT) Department, Attikon University Hospital, National and Kapodistrian University of Athens, Athens, GRC; 2 Medicine Department, Aristotle University of Thessaloniki, Thessaloniki, GRC

**Keywords:** bisphosphonates, sodium fluoride, hearing loss, otospongiosis, otosclerosis

## Abstract

Otosclerosis, also known as otospongiosis, is a primary osteodystrophy of the otic capsule of the inner ear and one of the leading causes of deafness in adults. The rationale for medical therapy for otospongiosis is to slow down and eventually stop the phase of bone resorption. Conservative treatments include sodium fluoride (NaF), bisphosphonates, and other modern medicines. A systematic review of the existing and published articles and books until April 2021 has been conducted in Medscape, Google Scholar, PubMed, and other databases using appropriate terms. According to the results of the research, the administration of NaF for a period of at least six months stabilizes hearing thresholds (HTs), improves vestibular symptoms, and delays the worsening of tinnitus. The administration of bisphosphonates for a period of at least six months showed significant percentage differences in the improvement of hearing loss, dizziness, and tinnitus remission. In the already existing double-blind studies that were evaluated, groups of patients treated with bisphosphonates for at least 24 months showed greater stabilization of the mean air and bone conduction thresholds than groups of patients treated with a placebo. The new modern medications have not yet been widely administered clinically to draw useful conclusions, although the test results of some of their use are quite encouraging.

## Introduction and background

The term otosclerosis is used to describe the primary disease of the temporal bone localized in the bony capsule of the labyrinth, which is characterized by alternating phases of resorption and formation of bone tissue. In otosclerosis, mature normal solid bone tissue is degraded by osteoclasts and replaced by newly formed bone tissue produced by osteoblasts. The location of otosclerotic foci may be located in different parts of the bony labyrinth [1,2].

The Italian anatomist Antonio Maria Valsalva was the first to describe the lesions of otosclerosis by performing an autopsy on a deaf person in the 18th century [3]. The term otosclerosis was first formally used in 1984 by Politzer to describe the final inactive stage of bone remodeling in which the bone has become solid and sclerotic [4]. The term “otospongiosis,” first used by Siebenmann in 1912, refers to the active and vascular stage of the process of bone remodeling and is more accurate from a pathological point of view as it indicates the active phase of the lesion [5]. In 1873, Schwartze was the first to describe the reddish hue of the tympanic membrane otoscopically [3]. Julius Lampert in 1932 published his technique for the surgical treatment of the condition, followed by John Shea, who was the first to describe the innovative technique of anovectomy, replacing the stapes with the addition of a plastic polyethylene graft [6].

Etiopathogenesis

The prevalence of otosclerosis shows geographical variation and significant differences between different populations [7]. Otosclerosis is a multifactorial disease, which progressively leads to the development of hearing loss and for which several theories have been proposed regarding its pathogenesis. Although it has been the focus of many studies in recent decades, the factors contributing to the onset and progression of the disease are not fully clear, and the main ones that have been associated are as follows: (a) genetic factors, (b) viral infections, (c) autoimmune mechanisms, (d) cytokines, and (e) hormonal factors [8-17].

The main symptom of otosclerosis is conductive hearing loss, which occurs most frequently between the third and fifth decade of adulthood. Of patients who will develop conductive hearing loss, a proportion of 10% will also develop sensorineural hearing loss [18]. Affecting only the cochlea and developing sensorineural hearing loss are quite rare [19]. Hearing loss is perceived by the patient when the hearing threshold (HT) exceeds 25-30 dB, at which the patient has difficulty understanding speech [20]. Many patients with otosclerosis describe an improvement in their hearing in noisy environments, a phenomenon known as Willis paracusis [21]. As many as 56%-84.5% of patients with otosclerosis experience tinnitus, particularly in the cochlear type of otosclerosis [22,23]. Vestibular symptoms, most commonly benign paroxysmal positional vertigo, balance disorders, and dizziness, have been reported in up to 10%-30% of patients with otosclerosis, but their pathophysiology remains unclear [11].

Possible therapeutic targets

In recent decades, the molecular mechanisms underlying bone metabolism have been the focus of research [2,24,25]. Bone metabolism is under dual regulation. In addition to the known influence of the endocrine system on bone metabolism, a local network of a complex interplay between osteoblasts, osteoclasts, and many other mediators has also been identified while the balance between resorption and new tissue formation is regulated by cytokines as well as other mediators described below [26].

The cytokine receptor activator of nuclear factor kappa B ligand (RANKL) is secreted by a variety of cells including osteoblasts. This cytokine induces differentiation, activation, and survival of osteoclasts as it activates the osteoclast-specific receptor RANK [27]. Osteoprotegerin (OPG) is a potent inhibitor of bone resorption and reduces osteoclast activity. Osteoprotegerin is a soluble receptor that acts antagonistically toward the osteoclast RANK receptor by binding the cytokine RANKL and thereby inhibits the differentiation, activation, and survival of osteoclasts and promotes their apoptosis [27-30]. In patients with otosclerosis, high levels of OPG mRNA were detected in the helical ligament, and high concentrations of OPG cytokine were detected in the periosteum, while in contrast, no levels of OPG were detected within the bony otic capsule by immunohistochemistry technique [29,30]. Thus, osteoprotegerin may be produced by the soft tissues of the cochlea and diffused into the periosteum and periosteal structures. This is likely one of the reasons why the otic capsule’s morphology and development are unique. Normal otic capsule exerts minimal bone turnover and contains almost no osteoclasts [31].

The activity of an otosclerotic foci can be classified by category, starting from category I (fully active) to category IV (fully inactive), depending on cytobrism, angiobrism, percentage of extracellular collagen fibers, and presence of osteoblasts and osteoclasts [14]. There are many osteoclasts, fibroblasts, giant cells, and proliferating endothelial cells in active otosclerotic foci, which may be responsible for the increased metabolic activity and the development of spongiotic damage that ultimately leads to bone resorption. In response to this enhanced bone resorption, a regenerative process occurs in the foci, leading to their fibrous transformation by osteoblasts and fibroblasts [32,33].

The early stages of otosclerosis have been associated with the presence of inflammation due to measles virus infection. The active phase of the disease was characterized by increased inflammatory status, the presence of measles virus particles, abundant local expression of tumor necrosis factor-α (TNF-α), and the absence of osteoprotegerin. The dormant phase was characterized by the presence of osteoprotegerin and measles virus simultaneously with the absence of TNF-α and the absence of inflammation [12,34,35]. In the early stages of otosclerosis, the detection of TNF-α correlates with the expression levels of measles virus RNA fragments [33]. Measles virus particle antigens are expressed on the surface of infected cells by major histocompatibility complex class I (MHC-I) molecules in osteoblasts, fibroblasts, and endothelial cells. Thus, the CD8 T lymphocyte-dependent immune response leads to the release of TNF-α and subsequently to bone resorption.

Medication

The therapeutic approach chosen for the treatment of otosclerosis depends on the patient’s symptoms, general health, age, degree and type of hearing loss, and bilateral or unilateral location of the lesion. Treatment options consist of either simple monitoring, hearing aids, surgical treatment, or medical treatment of the disease [19,36-40].

Surgery is the gold standard treatment for otosclerosis, but it may not result in full recovery of patients or complete symptom alleviation. Considering also the viral and autoimmune inflammatory nature of the pathogenesis of otosclerosis leading to abnormal bone remodeling, anti-inflammatory, anti-osteoporotic, and other pharmaceutical agents can be used to treat the disease. To date, there is no therapeutic treatment for otosclerosis, and the research undertaken has focused on the efficacy of medication in cases of active otosclerosis to reduce the disease’s progressive degenerative activity. The more widely accepted enzymatic theory attempts to explain the harm induced by otosponsification to the inner ear. The utilization of bone metabolism inhibitors is intended to preserve the auditory cords (neurosensory component) and alleviate symptoms including tinnitus and vertigo. For the treatment of otosclerosis, sodium fluoride (NaF), bisphosphonates, and other formulations such as bioflavonoids, vitamin D, vitamin A, corticosteroids, nonsteroidal anti-inflammatory drugs (NSAIDs), immunosuppressive agents, and biological treatments are available.

## Review

Methods

A systematic review of existing published literature and articles up to January 2022 in the PubMed database was conducted by searching for appropriate terms. The keywords used were “medical management of otosclerosis,” “sodium fluoride in otosclerosis treatment,” “bisphosphonates for otosclerosis,” “otosclerosis,” “otospongiosis,” “conservative treatment,” and “pharmacological treatment” for otosclerosis and/or otospongiosis in various combinations.

With regard to sodium fluoride treatment, the search criteria included the following: (a) the age of the patients to be over 18 years old, (b) patients who were treated with sodium fluoride not to have undergone surgical rehabilitation during the follow-up of the treatment results, (c) the exact dosage and time interval of the medication, (d) the follow-up of the patients by performing regular neuro-otological checkups, and (e) the lack of data from each study not to exceed 10%. With regard to bisphosphonates and other modern formulations, the search criteria included the following: (a) the age of the patient to be over 18 years old, (b) the follow-up of the treatment results to be at least six months, and (c) the lack of data from each study not to exceed 10%.

**Figure 1:**
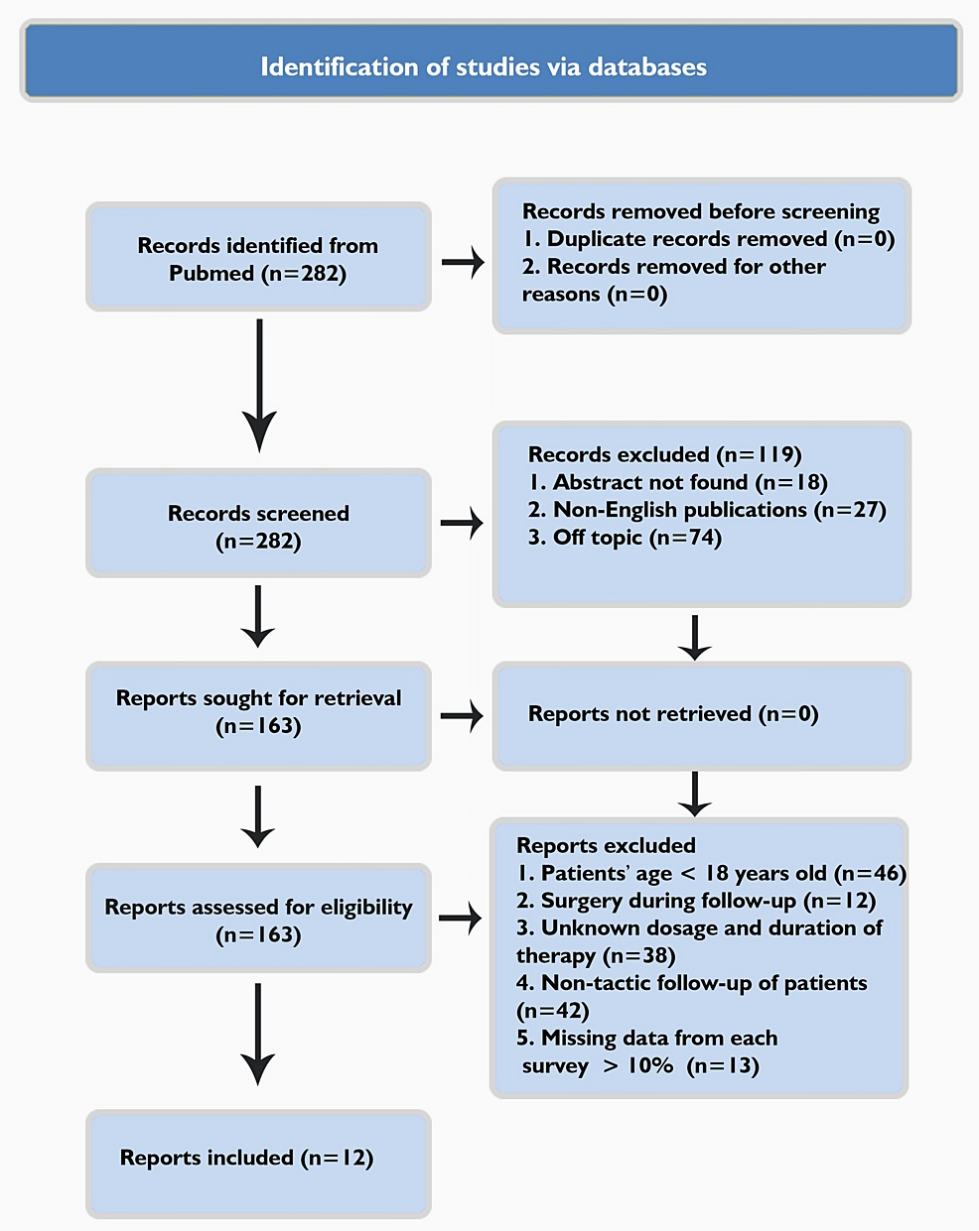
PRISMA flowchart of the search for conservative otosclerosis treatment PRISMA: Preferred Reporting Items for Systematic Reviews and Meta-Analyses

A total of 282 articles were found, from which, after a relevant study of their abstracts, those related to the drug treatment of otosclerosis with sodium fluoride and other modern formulations were retrieved. In addition, the references of these articles were assessed to further identify potentially relevant publications. Studies lacking details on treatment and outcomes, as well as articles whose full text was not accessible, were excluded. Articles in languages other than English such as Dutch, French, or German were also excluded (Figure 1).

Results

Twelve articles were included in this study, nine on the use of sodium fluoride (NaF) and three on the use of bisphosphonates (Table 1 and Table 2) [41-52].

**Table 1:** Characteristics of studies on patients with otosclerosis treated with NaF NaF: sodium fluoride, P: placebo, NT: no treatment, Glu Ca: calcium gluconate

Study	Number of participants	Number of patients treated with NaF	Number of patients treated with P or NT	Dosage (mg/day)	Duration (months)	Improvement or stabilization of hearing loss (ΝaF (%)/P (%) or NT (%))	Deterioration of hearing loss (ΝaF (%)/P (%) or NT (%))	Coadministration of other medication	Follow-up > one year
Bretlau et al. [41]	95	43	52	40	12-24	93/75	7/25	Glu Ca (500 mg/day) plus vitamin D (400 mg/day)	Yes
de Oliveira Vicente et al. [42]	18	9	9	20	6	-	-	-	No
Ramsay et al. [43]	146	11 (ears)	NT (135 ears)	24	12-24	-	-	-	Yes
Félix-Trujillo et al. [44]	100	50	NT (50)	40	6	-	-	-	No
Shambaugh et al. [45]	1,436	1,402	NT (34)	40-60	6-96	83/32	17/68	-	No
Derks et al. [46]	41	19	NT (22)	75	12-60	-	-	-	No
Forquer et al. [47]	394	192	NT (202)	25	12-24	54/-	-	Glu Ca	No
Bébéar et al. [48]	40	40	-	60	6	-	-	-	No
Debry et al. [49]	136	89	NT (50)	15-60	1-6	-	-	-	No

**Table 2:** Characteristics of studies on patients with otosclerosis treated with bisphosphonates P: placebo

Study	Number of participants	Treatment	Follow-up (months)	Evolution of hearing-neurological symptoms		
				Improvement	Stabilization	Deterioration
Κennedy et al. [50]	26 (14 etidronate versus 12 P)	Etidronate 20 mg versus P	24	-	Control group > P group	-
Brookler et al. [51]	545	Etidronate	6-57	310	91	144
Jan et al. [52]	7 (14 ears)	*Risedronate* or zoledronate	89.2 (mean)	2 ears	11 (ears)	1 ear

Sodium Fluoride

Of the 95 patients in the study of Bretlau et al., a statistically significant difference (p<0.025) was found in the worsening of hearing loss (>10 dB for at least one of the frequencies of 500 Hz, 1,000 Hz, and 2,000 Hz) between the sodium fluoride group and the placebo group [41].

The study of de Oliveira Vicente et al. compared the treatment results in patients with otosclerosis before and after the administration of NaF or placebo medication and showed that statistically significant differences were mainly in the high frequencies (2,000 Hz and 4,000 Hz) [42].

The analysis of the acoustic data of the right ears of the two groups in the paper by Derks et al. showed that patients in the NaF group showed a mean deterioration of bone conduction values of 1.37 dB compared to 9.54 dB for the untreated patients (p<0.001). Regarding the 2,000 Hz and 4,000 Hz frequencies, the mean deterioration in bone conduction in the NaF group was 0.15 dB compared to 8.18 dB in the untreated patient group (p<0.008) [46].

In a study including 192 patients who received 25 mg of NaF and calcium daily for two years, Forquer et al. found that sodium fluoride slowed or reversed the progression of hearing loss in 104 patients. Of these patients, 63% had cochlear otosclerosis and 46% had otosclerosis with stapes base fixation (p<0.02) [47].

In the work of Shambaugh and Causse, NaF was administered to patients with active otosclerosis of the otic capsule at a moderate dosage (40-60 mg daily) for up to 10 years. The control group who had confirmed surgical otospongiosis with stapes fixation and did not receive the treatment showed a much faster progression of hearing loss than those treated with sodium fluoride [45].

Causse et al. in their study of 224 patients found an improvement in nystagmus recordings on electronystagmography that even approached normal recordings at almost 70% in cases of operated ears and 60% in cases of non-operated ears, compared with control groups, after NaF treatment for six months or more [53].

Bisphosphonates

Kennedy et al., in their study regarding the hearing pathways in patients with otosclerosis after the administration of etidronate (20 mg daily) for 24 months, reported a tendency to stabilize the progressive deterioration of hearing loss seen in otosclerosis. The placebo group has “worse” air conduction than the etidronate group, but with no statistically significant difference between the two groups [50].

Brookler and Tanyeri evaluated the effectiveness of etidronate as a treatment for the symptoms caused by otosclerosis in the inner ear. They analyzed the data of patients who had been diagnosed with otosclerosis with symptoms of dizziness, hearing loss, tinnitus, or Meniere’s syndrome. The study included 545 patients who had been treated with etidronate for more than six months. At follow-up, 54% of patients reported a subjective improvement in dizziness, and 39% of patients reported that dizziness disappeared. Regarding hearing loss, the results showed that hearing remained the same in 71% of cases and improved in 27% of patients. As for tinnitus, it improved in 52% of cases and disappeared completely in 16%. Finally, with regard to the responses of patients suffering from Meniere’s disease, 55% of patients reported complete relief of symptoms [51].

According to the results of Jan et al., patients with cochlear otosclerosis treated with third-generation bisphosphonates showed stabilization of their progressive sensorineural hearing loss at long-term follow-up [52].

Discussion

All patients with clinical signs of otosponsification should consider pharmacological therapy, which, according to the results of the present literature, can range from an adjuvant treatment to surgery or a hearing aid to the primary treatment choice for disease management.

Sodium Fluoride

The studies included in this review show that in patients administered NaF, there is an improvement in mean hearing loss at 2,000 Hz and 4,000 Hz, it is most effective when the initial sensorineural hearing loss is <50 dB, and by administering NaF 75 mg daily to patients with cochlear otosclerosis, the progression of the condition can be slowed down with regard to both low and high frequencies of bone scar of the tonal audiogram [41,42,46].

The shortcomings of these studies do not negate their usefulness. For example, the weakness of the study by de Oliveira Vicente et al. is that the disease based on the initial air and bone scores was more severe in the group of patients receiving placebo medication, whereas in the study by Causse et al., there is no statistical data analysis of the results reported, and the authors did not reveal the total number of people in the control group [42,53]. Nevertheless, the results continue to be remarkable as they show that NaF treatment can be successful for almost 80% of patients [47]. The findings of the above studies suggest that patients with faster rates of hearing loss progression responded more favorably to NaF treatment; hence, patients with the most active otospongeal lesions are more sensitive to treatment.

A question in the literature is the duration of NaF treatment. Shambaugh and Causse reported that when NaF administration was discontinued, a reactivation of the otosclerotic focus was observed in 8% of the patients 2-7 years later [45]. For this reason, for these patients, it was suggested to continue 20 mg of lifetime NaF administration to prevent reactivation of the otosclerotic focus. On the other hand, Derks et al. reported that two years of treatment did not appear superior to long-term treatment of four years [46]. Although it is commonly accepted that tonal audiometry findings show a greater tendency for stabilization of the air and bone conduction cords during the six months of sodium fluoride treatment, it should be noted that the six-month treatment duration may not have been sufficient to bring about significant improvement or stabilization of the hearing cords on various audiological tests.

Our review confirms the conclusion that after a few months of treatment with sodium fluoride, a remission of tinnitus intensity is reported. This is suggested by the results of the study by Causse et al. and Cody and Baker, who reported that after the administration of NaF in combination with calcium gluconate and vitamin D, recurrent attacks of vertigo were controlled or reduced in a large percentage of patients [53,54].

Bisphosphonates

The results of our study show that etidronate may have a positive effect on hearing stabilization in patients with worsening hearing loss due to otosclerosis and third-generation bisphosphonates have an important role in the treatment of patients with cochlear otosclerosis who have progressive sensorineural hearing loss.

Newer generations of bisphosphonates are regarded as more powerful bone resorption inhibitors [31]. Among the most potent last-generation bisphosphonates that inhibit bone resorption are those containing nitrogen atoms in a heterocyclic ring, such as risedronate and zoledronate, the latter with up to a thousand times the potency of etidronate, a more favorable tolerability profile, and the advantage of intravenous administration [55,56]. Bisphosphonates decrease bone resorption by interfering with the metabolism of osteoclasts and causing their apoptosis.

Through the same process, the production of toxic enzymes is secondarily reduced, followed by their dissemination into the peri-myxoma [31]. For alendronate, which is a bisphosphonate that has a similar action to etidronate, it is recommended to be administered in a daily dose of 10 mg. Risedronate is recommended to be administered at a dose of 5 mg per day. Formulations with a higher concentration of alendronate and risedronate may be administered once a week at a dosage of 70 and 35 mg, respectively. Also, bisphosphonates such as clodronate, pamidronate, and zoledronate may also be administered intravenously in doses of 1,500 mg (monthly), 90 mg (monthly), or 4 mg (yearly), respectively, with an infusion of four hours for clodronate and pamidronate and at least 15 minutes for zoledronate [25,57,58]. Third-generation bisphosphonates such as risedronate and zoledronate, alone or in combination, have also been proposed for the treatment of sensorineural hearing loss associated with otosclerosis [59]. Treatment with bisphosphonates has also been suggested in cases where sodium fluoride treatment has not been well tolerated [25].

Lack of patient adherence to therapy is one of the biggest issues with the use of bisphosphonates, primarily due to the expensive cost of the prescription and, in some cases, unpleasant effects. Alendronate use for more than three years has been connected with an increase in atypical femoral fractures, which occur in the absence of trauma [55]. Current perspectives and randomized studies conducted have paved the way for their administration in cases of otosclerosis.

Bioflavonoids

Studies have reported improvement in the symptoms of hearing loss and tinnitus due to otosclerosis, both preoperatively and postoperatively, following the administration of bioflavonoids for a period of approximately six months. More specifically, Sziklai et al. indicated that after six months of treatment with 7-isopropoxy-isoflavone (bioflavonoid) for tinnitus caused by otosclerosis, all patients had improved tinnitus, whereas only 50% of placebo patients did. However, this study has poor power due to the small number of patients enrolled in the study. Further research is needed [60].

Vitamin D

Vitamin D has also been associated with the development of inflammation and autoimmunity, in addition to bone metabolism. Vitamin D deficiency has been linked to a number of autoimmune disorders [61]. As an additional therapy for autoimmune illnesses, vitamin D supplementation has been evaluated. In addition to its effects on bone metabolism, vitamin D supplementation in otosclerosis may also be beneficial due to its anti-inflammatory effects [26]. Furthermore, hypovitaminosis D has been associated with the progression of otosclerosis [62]. From a review of the current literature, only one study was found that included 47 patients with otosclerosis in which the possible etiological role of vitamin D in the diet was investigated. Three of the 16 individuals who took vitamin D and calcium supplements experienced a considerable improvement in their hearing as a result of the treatment.

Vitamin A

Although no work was found on vitamin A, it is worth mentioning that hypervitaminosis A tends to accelerate the “thinning” of long bones and the thinning of their bone density, by increasing fragility and causing spontaneous fractures in experiments carried out in animal models. This, through still-unknown mechanisms, may be attributable to osteoclast-mediated bone resorption [63,64]. During in vitro studies in humans and mice, retinoic acid increased the proliferation of osteoclast progenitor cells and inhibited RANK-induced differentiation of osteoclasts. According to the majority of publications obtained from bone marrow studies, retinoic acid can probably inhibit osteoclast differentiation, so especially in cases of vitamin A deficiency, its administration as a dietary supplement may prevent the onset of the disease [47].

Anti-inflammatory Agents

Systemic and intratympanic administration of corticosteroids: The early active phase of otosclerosis may be caused by osteolytic inflammation associated with measles virus infection and/or an autoimmune anti-collagen type II reaction. These processes may justify the use of anti-inflammatory drugs in otosclerosis. Reduced inflammation is the result of corticosteroid treatment, which inhibits the production of inflammatory cytokines and growth factors [65]. A characteristic feature is an increase in the concentration of corticosteroids in the periosteum of patients receiving intravenous corticosteroids (prednisolone 250 mg). Local administration of corticosteroids in the inner ear may be a beneficial alternative that will not cause the systemic adverse effects of corticosteroids [66]. Intravenously administered glucocorticoids pass through the oval follicle and enter the periosteum, thus avoiding systemic adverse effects and not causing damage to the cochlear hair cells. In a study in which five intratympanic injections of 4 mg/mL of dexamethasone were administered, no adverse effects on outer hair cell function were observed, as demonstrated by transiently evoked otoacoustic emissions. Thus, intratympanic administration of dexamethasone appears to be safe [26,67].

Nonsteroidal anti-inflammatory drugs (NSAIDs): NSAIDs work by blocking the enzyme cyclooxygenase, which is essential for the production of prostaglandins (Pgs) from the arachidonic acid [68,69]. Prostaglandins have pleiotropic effects on bone, being able to promote both bone tissue resorption and new bone formation in vitro and in vivo. It is possible that prostaglandins and their inhibitors can influence the process of bone tissue resorption that occurs in otosclerosis. The inhibitory effect, in terms of bone resorption of indomethacin and calcitonin, on lesions similar to those of otosclerosis in mice has also been described, as has the reduction in osteoclast number following the administration of indomethacin in healthy animal models. However, there are no data available regarding the long-term application of NSAIDs.

Immunosuppressive Agents

Although the inflammatory nature of otosclerosis, as well as the involvement of multiple inflammatory cells and mediators, at least in the early phase of the disease, has been well elucidated, there are no available reports on the use of immunosuppressive drugs such as methotrexate, leflunomide, azathioprine, or cyclophosphamide in this disease. The only immunosuppressive agent whose use has been documented is cyclosporine A [70].

Possibilities of Targeted (Biological) Treatments

Anti-TNF-α agents: TNF-α is an important regulator of bone remodeling, which is abundantly produced in the active phase of otosclerosis [30,33]. Local expression of the TNF-α factor in the otic capsule can trigger the inflammatory response and bone resorption seen in otosclerosis; therefore, local or systemic administration of TNF-α inhibitors may be an option for the medical management of otosclerosis and its associated sensorineural hearing loss [24]. Intratympanic administration of infliximab has shown positive results in only one pilot research including individuals with sensorineural hearing loss [71].

Recombinant osteoprotegerin: OPG is a potent inhibitor of bone resorption, as it reduces osteoclast activity by decreasing osteoclast production and indirectly interfering with TNF-α-mediated RANK-RANKL interaction [29]. Because the active phase of the disease is characterized by abundant expression of TNF-α and the absence of osteoprotegerin, the administration of exogenous OPG could inhibit bone disorganization performed by osteoclasts [35]. The use of recombinant human OPG (rhOPG) has been started in animal research; however, there are no reports from humans as of yet [72].

Prospects for other anti-osteoporotic targeted therapies in otosclerosis: Denosumab is a human monoclonal antibody that binds to RANKL, preventing the activation of its receptor, RANK, on the surface of osteoclast precursors and also mature osteoclasts. The inhibition of the RANKL-RANK interaction inhibits the formation, function, and survival of osteoclasts, thereby reducing bone resorption in both spongy and solid bone. Denosumab has been approved for the treatment of postmenopausal osteoporosis and may potentially be applied to otosclerosis in the future if there are sufficient long-term studies demonstrating the benefits of the treatment in patients with otosclerosis [73].

Measles vaccination: Infection with the measles virus is a major risk factor for the onset of otosclerosis. In particular, the finding of measles virus-specific IgG antibodies found in the pericytes of people with otosclerosis supports the involvement of the virus in the development of the disease [8]. Studies have shown that vaccination against the measles virus has reduced the incidence of patients with otosclerosis and increased the average age of onset of the disease, slowing its progression. This demonstrates and emphasizes the need for measles vaccination, especially in countries where the vaccine is not included in national vaccination programs, to prevent the onset of the disease by combating an important causative agent [14,74].

Finally, current data indicates that traditional stapedotomy remains the gold standard for the treatment of otosclerosis, while endoscopic cochlear implantation is a viable option for advanced mixed or sensorineural hearing loss cases [75,76].

## Conclusions

The efficacy of medication in cases of active otosclerosis to reduce the disease’s progressive degenerative activity is well documented, while noninvasive treatments should be prescribed even in surgical cases since surgery does not etiologically influence the progression of the disease. Sodium fluoride and bisphosphonates are the main treatments currently used to prevent the onset or progression of otosclerosis symptoms. NaF, in combination with calcium carbonate and vitamin D, can reduce the rate of deterioration of both conductive and sensorineural hearing loss due to otosclerosis. Beneficial is the effect of NaF on the control of vestibular symptomatology in otosclerotic patients and on the worsening of tinnitus. NaF treatment at lower doses also appears to be effective in inhibiting the progression of the disease in its early stages. However, so far, there are no clear and precise indications as to how long NaF treatment should be continued.

Particularly promising in the treatment of otosclerosis are the bisphosphonates, which are gaining ground, judging by not only the results of their action in the treatment of patients with otosclerosis but also by the possibilities of different routes of administration (oral or intravenous) and the frequency of doses (daily, monthly, or annually). They can be used as a substitute for NaF if the patient is intolerant of or has a contraindication to NaF treatment, or as an adjuvant to NaF treatment. Other modern formulations, many of which are still in the research stage, may be administered for the treatment of hearing loss or other symptoms due to otosclerosis in combination with fluoride and bisphosphonate-based medicines.
